# Using item response theory (IRT) to improve the efficiency of the Simple Clinical Colitis Activity Index (SCCAI) for patients with ulcerative colitis

**DOI:** 10.1186/s12876-021-01621-y

**Published:** 2021-03-22

**Authors:** Alissa Walsh, Rena Cao, Darren Wong, Ramona Kantschuster, Lawrence Matini, Jean Wilson, Andrey Kormilitzin, Matthew South, Simon Travis, Sarah Bauermeister

**Affiliations:** 1 Translational Gastroenterology Unit, NIHR Oxford Biomedical Research Centre, Nuffield Department of Experimental Medicine, University of Oxford, John Radcliffe Hospital, Oxford, UK; 2 Department of Psychiatry, University of Oxford, Oxford, UK

**Keywords:** Ulcerative colitis, Activity index, Item response theory

## Abstract

**Background:**

The SCCAI was designed to facilitate assessment of disease activity in ulcerative colitis (UC). We aimed to interrogate the metric properties of individual items of the SCCAI using item response theory (IRT) analysis, to simplify and improve its performance.

**Methods:**

The original 9-item SCCAI was collected through TrueColours, a real-time software platform which allows remote entry and monitoring of patients with UC. Data were securely uploaded onto Dementias Platform UK Data Portal, where they were analysed in Stata 16.1 SE. A 2-parameter (2-PL) logistic IRT model was estimated to evaluate each item of the SCCAI for its informativeness (discrimination). A revised scale was generated and re-assessed following systematic removal of items.

**Results:**

SCCAI data for 516 UC patients (41 years, SD = 15) treated in Oxford were examined. After initial item deletion (Erythema nodosum, Pyoderma gangrenosum), a 7-item scale was estimated. Discrimination values (information) ranged from 0.41 to 2.52 indicating selected item inefficiency with three items < 1.70 which is a suggested discriminatory value for optimal efficiency. Systematic item deletions found that a 4-item scale (bowel frequency day; bowel frequency nocturnal; urgency to defaecation; rectal bleeding) was more informative and discriminatory of trait severity (discrimination values of 1.50 to 2.78). The 4-item scale possesses higher scalability and unidimensionality, suggesting that the responses to items are either direct endorsement (patient selection by symptom) or non-endorsement of the trait (disease activity).

**Conclusion:**

Reduction of the SCCAI from the original 9-item scale to a 4-item scale provides optimum trait information that will minimise response burden. This new 4-item scale needs validation against other measures of disease activity such as faecal calprotectin, endoscopy and histopathology.

## Background

The development of accurate measures for assessing disease activity in UC is crucial to the longitudinal evaluation of patient response to treatment in a treat-to-target approach [1].There is growing interest in using electronic patient reported outcomes (ePROs) in both routine clinical care and clinical trials as a means to facilitate patient-centred care. In addition, ePROs have demonstrated enhanced patient satisfaction, efficiency of standardised assessments and completeness of data collection when compared to paper-based patient-reported outcome measures [2, 3]. When implementing such data collection systems, response burden must be considered. This is defined as the degree of effort required for patient to complete a set of questionnaires and matters because of correlations between response burden and response rates, completion rates and data quality [4, 5]. Questionnaire length and frequency increase response burden [6]. Questionnaires need to be streamlined to reduce response burden without compromising the precision of the data.

The Simple Clinical Colitis Activity Index (SCCAI, Table 1) was designed in 1998 to facilitate accurate assessment of disease activity in UC without physical, laboratory, or endoscopic indices [7]. The SCCAI was designed before contemporary criteria for index development existed and remains to be fully validated, but is widely used in clinical practice or clinical trials [8], with evidence of robust discriminative and construct validity [9, 10], both as a physician- and patient-administered questionnaire [11, 12]. Nevertheless, shortcomings immediately became apparent: whilst correlation coefficients between each item and the final SCCAI score ranged from 0.74 to 0.80, the correlation coefficient for extraintestinal manifestations (EIMs) of UC had a correlation coefficient of 0.4. The reason for including EIMs in the SCCAI was historical, since it had been thought that some were a feature of disease activity. The poor correlation suggests a low likelihood of discriminative information, suggesting a dissociation between the EIMs and the rest of the scale items.

**Table 1:** Original simple clinical colitis activity index

Item	Scale	Score
Daytime frequency	0–3	0
	04-Jun	1
	07-Sep	2
	> 9	3
Nocturnal frequency	0	0
	01-Mar	1
	04-Jun	2
Urgency of defaecation	None	0
	Hurry	1
	Immediately (toilet nearby)	2
	Incontinence	3
Blood in stool	None	0
	Trace	1
	Occasionally frank (< 50% of stool)	2
	Usually frank (> 50% of stool)	3
General well-being	Very well	0
	Slightly below par	1
	Poor	2
	Very poor	3
	Terrible	4
Extraintestinal manifestations (score 1 if present, 0 if not)	Arthritis	1
	Uveitis	1
	Erythema nodosum	1
	Pyoderma gangenosum	1

SCCAI ranges from 0 (best) to 19 (worst) [7]

Item Response Theory (IRT) is a statistical analysis technique used to assess and evaluate questionnaire-based measurement tools by including only those items with high discrimination that add to the precision of the tool and excluding those that have low discrimination. The technique has been widely implemented in the educational field for test development and has more recently been applied to developing, assessing and validating psychological scales, as well as patient-reported health outcomes [13]. We aimed to use IRT to interrogate the metric properties of individual items of the SCCAI and inter-item correlation, with the goal of identifying a reduced-item version with a reduced response burden that could be used for longitudinal assessment of patients with UC.

## Methods

### TrueColours

TrueColours [14] is a web-based, real-time software platform^i^ which allows remote entry and monitoring of patients with UC [15]. Through email prompts, linked to questionnaires, patients are able to record information relating to disease activity (SCCAI), quality of life, and outcomes such as steroid use and hospitalisation [15]. TrueColours is offered to all patients with UC being treated at the John Radcliffe Hospital in Oxford. All responses are held on a secure Oxford Health server. All patients were consented (written consent) to the Gastrointestinal Illness in Oxford: prospective cohort for outcomes, treatment, predictors and biobanking was approved by the NRES Committee Yorkshire and The Humber—Sheffield. REC Ref: 16/YH/0247. Date and Version No: 29/03/2019, Version 9, The University of Oxford is the sponsor. All data analysed was de-identified.

### Statistical methodology

Data were securely uploaded on the Dementias Platform UK (DPUK) Data Portal [16] where they were analysed using Stata 16.1 SE [17].

#### Summary statistics

Summary statistics are presented as mean (standard deviation) and median (IQR) for parametric and non-parametric data, respectively. Categorical data are presented as number (percentage).

#### Item response theory

*Theoretical basis* IRT encompasses a set of statistical modelling techniques that attempts to explain the relationship between the so-called ‘latent trait’ (in this case, ulcerative colitis disease activity) and their measured manifestations (in this case, each item of the SCCAI). Items should be scored in the same way (e.g., Likert or binary). Consequently, items 1–5 of the SCCAI were recoded as a binary score (Table 2), using the guidance of two experienced clinicians (AW, ST). This matched the scoring style of EIMs which were subsequently separated to create a 9-item scale suitable for mathematical analysis.

**Table 2:** Binary recoding schema of the SCCAI for IRT analysis

Items	SCCAI Score^a^		Binary score
*Item 1* Daytime frequency	0		0
	1–3		1
*Item 2* Nocturnal frequency	0		0
	1–2		1
*Item 3* Urgency	0–1		0
	2–3		1
*Item 4* Blood in stool	0		0
	1–3		1
*Item 5* General well-being	0–1		0
	2–4		1
*Item 6* Extra-intestinal manifestations	Arthritis (new Item 6)	Yes/no	1/0
	Uveitis (new Item 7)	Yes/no	1/0
	Erythema nodosum (new Item 8)	Yes/no	1/0
	Pyoderma gangrenosum (new Item 9)	Yes/no	1/0

*SCCAI* Simple Clinical Colitis Activity Index

^a^The SCCAI Score for each item is derived from Table 1

(i) Developed by the University of Oxford in partnership with Oxford Health NHS Foundation Trust with funding from the National Institute of Health Research.

IRT is also known as latent trait theory, where the model is dependent on a person’s latent trait (disease activity) and the probability that they will endorse specific items measuring that trait. There is an underlying assumption that every time a person responds to an item (endorsement or non-endorsement), they are providing information about their latent trait. There are two parameters of interest in IRT, difficulty (person or item level of latent trait) and discrimination (item trait information level). Difficulty is plotted on a standardised continuous scale, so that a positive difficulty means a person possesses the trait (that of disease activity in this case). Items are also placed on the same scale and a positive item difficulty is defined as the ability or strength of measuring the trait. Conversely, a negative difficulty for a person means the lack of trait possession, or for an item, it means lack of ability to measure the trait in that item. An example would be urgency: this is more likely to measure disease activity than physical strength and likewise will possess more information about disease activity, hence should have a positive difficulty value. The Item Information Function (IIF) curves display the amount of Information each item provides and its ability to measure that trait (difficulty).

Discrimination is plotted on Item Characteristic Curves (ICC), which are graphical representations of the relationship between the latent trait and item endorsement that are unique to IRT. A guideline discrimination (information) score > 1.7 is generally considered informative, but is scale-dependent, however this was used an approximate item elimination guide in this analysis [18]. By convention, scales expressing a range of latent trait values are more informative than items clustering around a single value. Discriminatory values are computed for item assessment (0.01 to 0.34 = very low; 0.35 to 0.64 = low; 0.0.65 to 1.34 = moderate; 1.35 to 1.69 = high;  > 1.70 = very high). Additional information about IRT may be found in the Additional file 1: S1.

#### Item response theory analysis

Measures of between-item relationships (scalability) and unidimensionality (single major trait) were estimated (see Additional file 1) prior to applying a 2-PL logistic IRT model across all 9 items. Since items 8 and 9 (erythema nodosum and pyoderma gangrenosum) did not vary in the estimation sample (i.e. item 8 endorsed 2/516 and item 9 endorsed 0/516 patients), they were subsequently eliminated from further analyses. The model was then estimated across the remaining 7 items.

#### Secondary analysis: scale revision

To evaluate a more efficient scale with improved item-information, low discriminatory items were removed from the scale. Items were removed by order of discrimination value, with the lowest discrimating item < 1.7 removed before the 2-PL model was re-estimated with the remaining items; the process was then repeated twice to remove poorly discriminating and inefficient scaling items. A 4-item scale suggested improved scaling and unidimensionality (see Additional file 1), and optimal informativeness and efficiency.

#### Reliability

We used Cronbach’s alpha (α) as a guide to measure whole scale reliability. Reliability is a measure suggesting the scale items are reliable at measuring an underlying construct or trait. Scales are considered reliable the higher the α score (i.e., 0.7—0.9 = good; 0.6 to 0.7 = acceptable; 0.5 to 0.6 = poor; < 0.5 = unaccetable) [19].

## Results

### Participants

Data from the baseline SCCAI questionnaire from 516 consecutive patients with UC were downloaded from the TrueColours platform. The population had a mean age of 42 years (SD = 15) and 281 (54%) were female. 24 (5%) were current smokers, 174 (34%) were ex-smokers and 318 (62%) had never smoked. Disease distribution was extensive in 155 (30%), 121 (23%) had left-sided disease, and 105 (20%) had proctitis.135 (26%) had an unknown distribution of disease. 35% of patients reported taking oral prednisolone in the last 12 months. The median duration of disease from diagnosis was 11 years (IQR 2.5–14.6 years).

### Analysis

#### Item scalability

The scales were assessed for unidimensionality (all items measure a single major trait construct) and item independence (items independently measure a single trait but are correlated to a limited extent). Further theoretical information is available in Additional file 1: S2. For the 7-item scale most items were significantly correlated (*p* < 0.01), with no items r > 0.50, suggesting that with the exceptions of items 6 and 7, each item measures a different trait symptom, suggesting basic item independence [20], yet maintaining measurement of a single underlying construct (see Additional file 1: Table S1). A factor analysis suggested a single major factor (trait) was correlated with items 1–4 but not 5–7, suggesting selective unidimensionality. Further details on the scalability and unidimensionality measures for the 7-item scale are presented in Additional file 1: S3.

#### 2-PL IRT

**Fig. 1:**
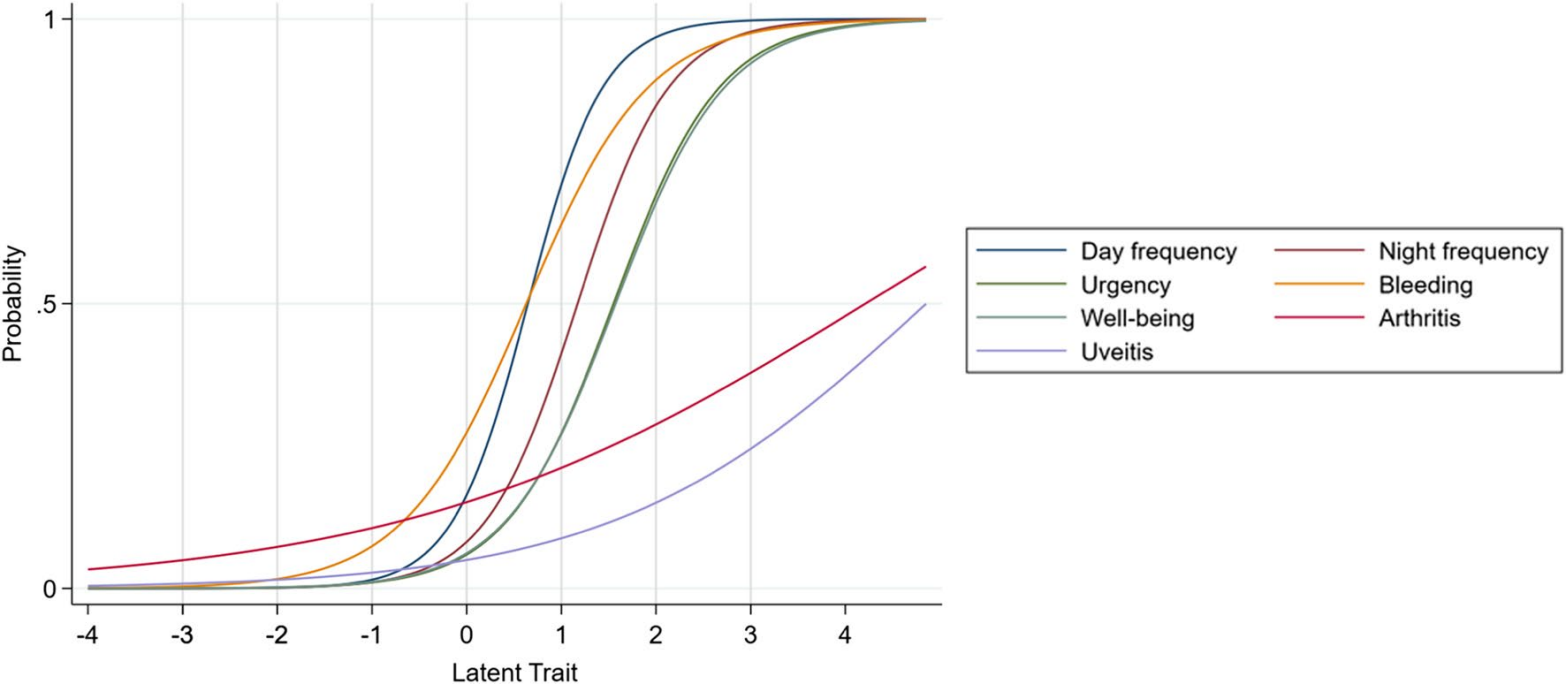
Item Characteristic Curves for the 7-item SCCAI Scale

Item parameter results of the 2-PL IRT 7-item scale are presented in Table 3. Figure 1 shows the ICC for each item. For example, for Item 1 (Daytime Frequency), there is a 50% chance that someone with a latent trait of 0.65 (broadly indicative of mild colitis symptoms) would endorse this item, so it is considered an item characteristic of colitis symptoms, albeit low.

**Table 3:** Item parameters for the 7-item and reduced 4-item 2-PL IRT models

Item		7-item					4-item				
		B^a^	SE^b^	Z^c^	^#^P	95% CI	B^a^	SE^b^	Z^c^	^#^P	95% CI
1	*Daytime freq*										
	Discrimination	2.52	0.47	5.37	0.000	1.60–3.44	2.78	0.47	5.89	0.00	1.85–3.70
	Difficulty	0.65	0.08	8.24	0.000	0.49–0.80	0.64	0.08	8.43	0.00	0.49–0.79
2	*Nocturnal freq*										
	Discrimination	2.07	0.35	5.96	0.00	1.39–2.75	2.16	0.37	5.83	0.00	1.43–2.88
	Difficulty	1.17	0.12	10.05	0.00	0.94–1.40	1.17	0.10	11.38	0.00	0.97–1.38
3	*Urgency*										
	Discrimination	1.78	0.31	5.69	0.00	1.17–2.39	1.52	0.29	5.32	0.00	0.96–2.08
	Difficulty	1.55	0.12	9.33	0.00	1.23–1.88	1.70	0.21	8.15	0.00	1.29–2.10
4	*Bleeding*										
	Discrimination	1.55	0.23	6.69	0.00	1.09–2.00	1.50	0.23	6.51	0.00	1.05–1.95
	Difficulty	0.63	0.10	6.52	0.00	0.44–0.82	0.65	0.10	6.57	0.00	0.46–0.85
5	*Well-being*										
	Discrimination	1.73	0.31	5.64	0.00	1.13–2.33					
	Difficulty	1.57	0.17	9.11	0.00	1.23–1.91					
6	*Arthritis*										
	Discrimination	0.41	0.16	2.53	0.01	0.09–0.73					
	Difficulty	4.21	1.58	2.65	0.01	1.10–7.32					
7	*Uveitis*										
	Discrimination	0.61	0.25	2.45	0.01	0.12–1.09					
	Difficulty	4.85	1.80	2.69	0.01	1.32–8.39					

^a^B = standard beta coefficient

^b^SE = standard error of estimation

^c^Z = z-score

^#^P = p > x (exact p-value)

**Fig. 2:**
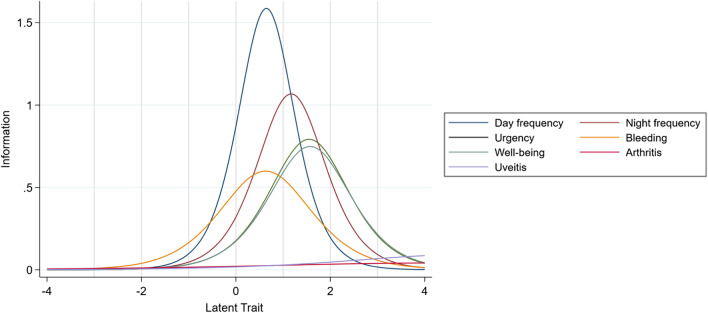
Item Information Curves for the 7-item SCCAI Scale

The IIF curves of each item are presented in Fig. 2. It is clear that items 6 (arthritis) and 7 (uveitis) possess very low information values (Table 3). Other items provide moderate to high levels of item information with discrimination values ranging from 1.55 to 2.52.

#### 7-item scale reliability

Overall scale reliability for the 7-item scale was acceptable (Cronbach α = 0.63).

#### Secondary analysis: scale revision

**Fig. 3:**
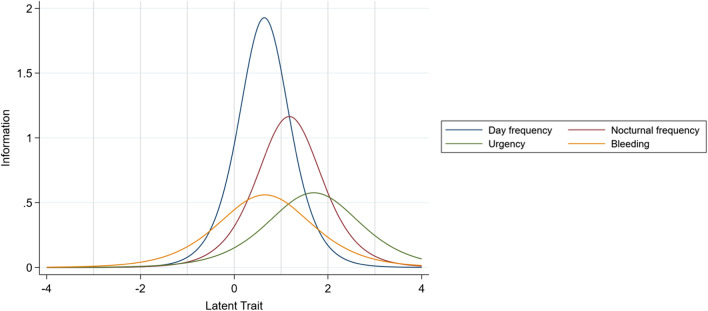
Item Information Curves for the revised 4-item SCCAI Scale

**Fig. 4:**
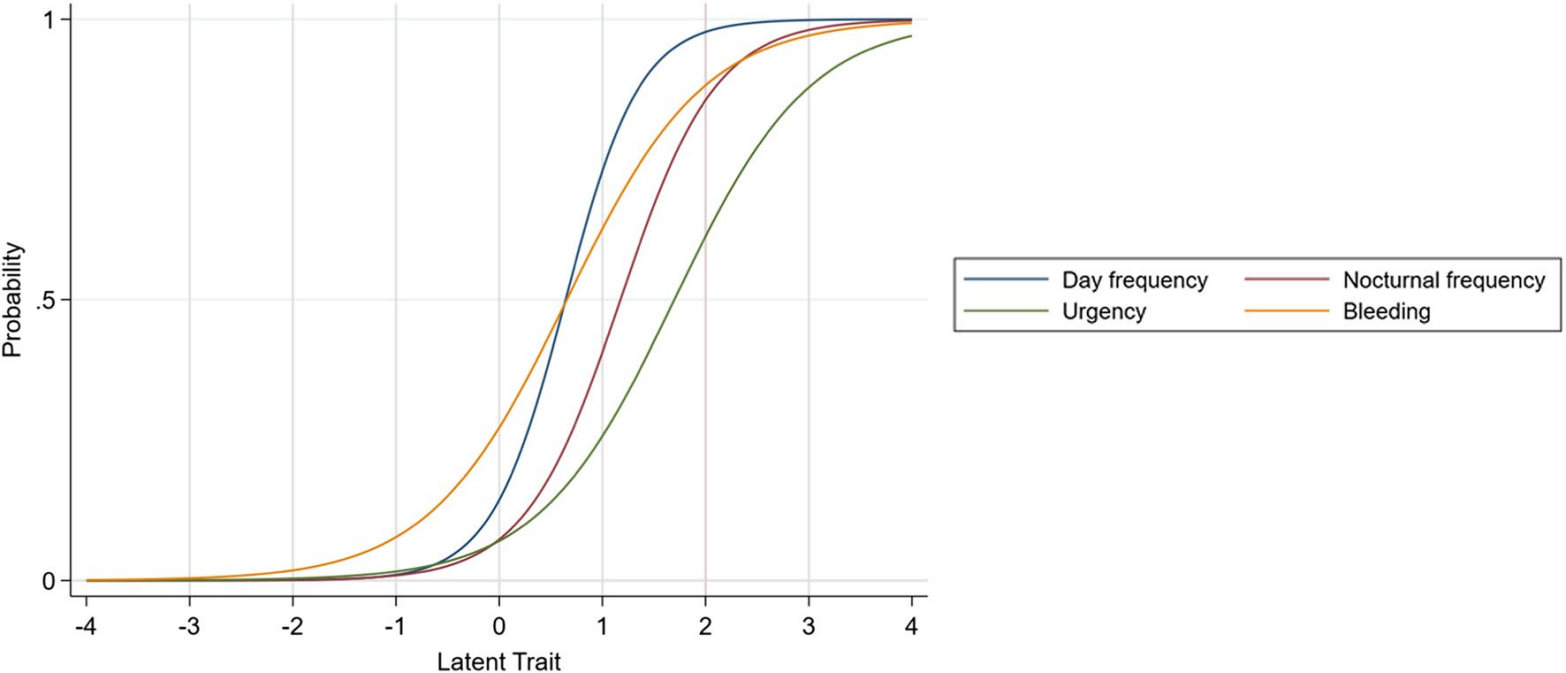
Item Characteristics Curves for the revised 4-item SCCAI Scale

Following the systematic removal of items through 2-PL IRT model, a 4-item scale was found to be more efficient, informative and with improved scalability and unidimensionality than the 7-item scale. All items in the 4-item scale were correlated (*p* < 0.01) with no items r > 0.5 suggesting basic item independence. A factor analysis suggested a single major factor was correlated with all items suggesting unidimensionality in the 4-item scale. Further details on unidimensionality and scalability measures for the 4-item scale are presented in Additional file 1: S4. The item parameters for the 4-item scale are presented in Table 3. The corresponding ICC and IIF graphs are presented in Figs. 3 and 4.

#### Reliability

Overall scale reliability for the 4-item scale is still low but higher than the 7-item scale (Cronbach α = 0.65).

## Discussion

This is the first study systematically to interrogate a commonly used clinical index for ulcerative colitis, using robust psychometric and statistical methodologies. The current 9-item SCCAI was found to have limited range, unscalable items and poor reliability, which is inefficient and likely to lead to redundancy between questionnaire items. When scale reductions were applied through a series of systematic revisions, it was found that a 4-item scale that included daytime frequency, nocturnal frequency, urgency and rectal bleeding provided the optimum trait information and scalability. Both the 7- and 4-item versions of the scale possessed weak reliability, but the 4-item scale was marginally more reliable. These findings imply that a reduced-item SCCAI would reduce response burden, whilst retaining maximal clinical information. Given that these questions are asked on a daily basis by the TrueColours platform, this reduction may enhance completion rates and sustain adherence to the programme. However, further work requires validation of the scale in another population to test current and additional items to assess clinical validation.

IRT has been successfully used to assess the efficiency of clinical measurement tools and subsequent item reduction, in a range of clinical contexts. It has been used to propose reductions in the burden of psychiatric evaluation tools, including the 19-item feelings scale for depression [21], the 16-item Anxiety Sensitivity Index [22] and 65-item Social Responsiveness Scale in autism spectrum disorder [23]. This methodology is increasingly adopted to modify indices measuring patient reported outcomes in chronic diseases, including the 11-item Roland-Morris disability scale for chronic pain [24], 14-item Valued Life Activities scale rheumatoid arthritis patients [25] and 31-item Qualeffo-31 for quality of life in osteoporosis [26]. All these studies have shown high correlation between the shortened index and the original, with regard to sensitivity, specificity and precision, despite the reduced number of items. One shortened scale demonstrated superior psychometric properties compared to the original [23].

A key strength of this study is the use of ePROs from the TrueColours’ database. This allowed a diverse, real-world sample of patients with differing levels of disease activity to be collected. This enabled IRT analysis to scrutinize the merit of each item in differentiating patients with inactive vs. severely active disease. Furthermore, rigorous quantitative analysis of the SCCAI of this large cohort permitted a sophisticated evaluation of an existing index constructed prior to the contemporary criteria for index development [27, 28].

Limitations to this study include that test–retest reliability was not measured, because each patients’ first response on the programme was the only one considered. Additionally, the binarisation of item responses has not been validated, even if it has clinical credibility. Furthermore, weak reliability was maintained at individual measures of the latent trait (disease activity) and overall using a 4-item scale, suggesting that the scale could benefit from the testing of revised items during validation. External validity of the reduced scale has yet to be tested, which is best achieved by comparing symptom responses measured by the modified SCCAI with more objective measures of disease activity, such as fecal calprotectin, endoscopic and histological disease activity.

Item response theory is a valid and robust psychometric methodology, which may be used to analyse patient-reported outcome questionnaires. We have shown that reduction of the SCCAI from the original 9-item to a 4-item scale provides optimum trait information. Changing the index would minimise the patient response burden in an era where ePROs are a pivotal component of improving outcomes.

Future aims would be to validate the 4-item scale against more objective markers of disease activity such as fecal calprotectin, endoscopy and histopathology.

## Conclusion

Our study demonstrated that the SCCAI could be reduced from the current 9-item scale to a 4-item scale—daytime frequency, nocturnal frequency, urgency and bleeding. Once validated, this reduced item, more efficient SCCAI would reduce response burden while retaining maximal clinical information. This is most important for programmes collecting longitudinal data as multiple response are required over extended time periods.

## Supplementary information

**Additional file 1.** Item Response Theory and Mathematical Assumptions of a 7-item Model.

## Data Availability

The datasets used and/or analysed during the current study are available from the corresponding author on reasonable request.

## Abbreviations

EIMs: Extraintestinal manifestations
ePROs: Electronic patient reported outcomes
ICC: Item Characteristic Curve
IIF: Item Information Function
IQR: Inter quartile range
IRT: Item Response Theory
SCCAI: Simple Clinical Colitis Activity Index
UC: Ulcerative colitis
