# Supplementary material for: Using item response theory (IRT) to improve the efficiency of the Simple Clinical Colitis Activity Index (SCCAI) for patients with ulcerative colitis
Source: BMC Gastroenterol. 2021 Mar 22;21:132. doi: 10.1186/s12876-021-01621-y (PMC7983213; doi:10.1186/s12876-021-01621-y)
Supplement: Supplementary file 1 — Additional file 1. Item Response Theory and Mathematical Assumptions of a 7-item Model. [file 12876_2021_1621_MOESM1_ESM.docx]

**Supplementary Materials**

**S1. Background Item Response Theory (IRT)**

IRT models describe the relationship between the trait or ability measured and response to the item. Traditionally, responses to scales are either dichotomous (two categories, i.e., yes or no) or polytomous (multiple categories, i.e., Likert scale). For this analysis we recoded the SCCAI into a 9-item binary response scale and a 2-parameter logistic (2-PL) IRT model is appropriate:

$$P\left( X_{i}=1 | \theta_{,}\beta_{i,} \alpha_{i} \right)= \frac{\exp(\alpha_{i} (\theta- \beta_{i}))}{1+\exp(\alpha_{i}(\theta- \beta_{i}))}$$

The dependent variable is the dichotomous response (yes/no) and independent variables are the person’s trait level (disease activity), theta (θ) and item difficulty ($\beta_{i})$. Independent variable scores combine cumulatively and the item’s difficulty is subtracted from θ. That is, the ratio of the probability of success for a person on an item vs. probability of failure, where a logistic function provides the probability that solving any item ($i)$ is independent from the outcome of any other item, controlling for person (θ) and item parameters (θ). The 2-PL model includes the two parameters representing item properties (difficulty and discrimination) in the exponential form of the logistic model.

**S2. Background mathematical assumptions**

Two major statistical assumptions are required for IRT parameter estimation, unidimensionality (all items measure a single trait construct) and item independence (items independently measure a single trait but are correlated to a limited extent) (DeMars, 2010). There is a selection of statistical tests which assess these assumptions and they are presented here in the context of this paper. Importantly, not all assumptions will be met adequately by the statistical tests and discretion is needed to assess the compliance to unidimensionality and item independence.

1. **Unidimensionality**

A fundamental assumption of an IRT model is that the response to an item is either a direct endorsement or non-endorsement of the trait (disease activity) measured and not a response to other factors. In other words, an individual’s response to a question is directly related to the level of the trait that they possess, suggesting a trait-specific unidimensionality (single dimension - in this case, disease activity) to the scale. Therefore, a scale or test that is unidimensional is expected to possess items which measure established components of a defined trait. No scale or test will have absolute unidimensionality, however, evidence that a main overall trait or factor is being measured can be computed through statistical procedures. A selection of these procedures is presented here to measure the assumption of unidimensionality.

Factor *analysis*

The first method is an inspection of the eigenvalues produced by a factor analysis to investigate the presence of a single major factor (unidimensionality). A promax rotation is applied, allowing factors to be correlated and aiding interpretation ^20^. The output can also be graphed and easily visualised using screeplot.

*Mokken analysis*

A further unidimensionality assumption of a scale is to assess the monotonicity of the scale. This assumption assesses the probability of a person endorsing an item measuring the trait means they are exhibiting or possessing characteristics of the trait. In other words, someone who has been defined as having ‘disease activity’ should be endorsing those items which reflect symptoms of the disease, i.e., ‘urgency’. The items therefore have to measure the underlying trait to an acceptable level. One method for assessing monotonicity is through a Mokken analysis wherein the Loevinger H coefficient is computed ^29^. This coefficient is a measure of the quality of a pair of items and can be expressed as a probability measure in which the ordering of the response probabilities of the items is independent of a respondent’s trait position (θ). For item pairs, *i* and *j*, *H_ij_* is defined as:

$$H_{ij}=\frac{P_{ij}- P_{i}P_{j}}{P_{i} (1- P_{j})} \left( P_{i}<P_{j} \right)$$

Where *P_i_* is in reference to the number of respondents who endorsed item *i* and *P_ij_* refers to the number of respondents who endorsed both items *i* and *j*. The *H* values produced by this analysis should ideally always be above .30 with a higher value suggesting a higher scalability (0.00 – 0.29 = none; 0.30 - 0.39 = weak; 0.40 – 0.49 = medium; 0.50+ = strong) ^30^

1. **Item independence**

In addition to the assumption of unidimensionality, it is a recommended that the items of the scale maintain local independence from each other and are not simply just rewordings of questions or duplications. A selection of procedures for assessing unidimensionality are presented here.

*Correlation analysis*

It is important that the items within a scale are correlated with each other but not ‘too highly’ (*r* < .50) suggesting basic item independence ^20^.

*Covariance residual coefficient correlation analysis*

A further measure of local independence measures is the assumption that once the dominant trait is controlled for (taking into account), the other items will no longer have high significant associations between each other. One method of assessing this is to examine the covariance residual correlation matrix produced after estimation of a single factor confirmatory factor analysis (CFA). High correlations (*r*  > 0.20) would indicate a local dependency between items ^31^. In other words, it would suggest that two items are measuring a similar characteristic of a trait and one of the items may be redundant or not adding any additional value to the scale in its existing format.

**S3. Mathematical assumptions 7-item model**

*Factor analysis*

A factor analysis was conducted and the outcome suggested that thre was a single major factor responsible for the majority of variance for items 1-4 but not 5-7. TA screeplot output is presented in Figure S1.

**Figure S1. Screeplot of the 7-item scale following a factor analysis**

*
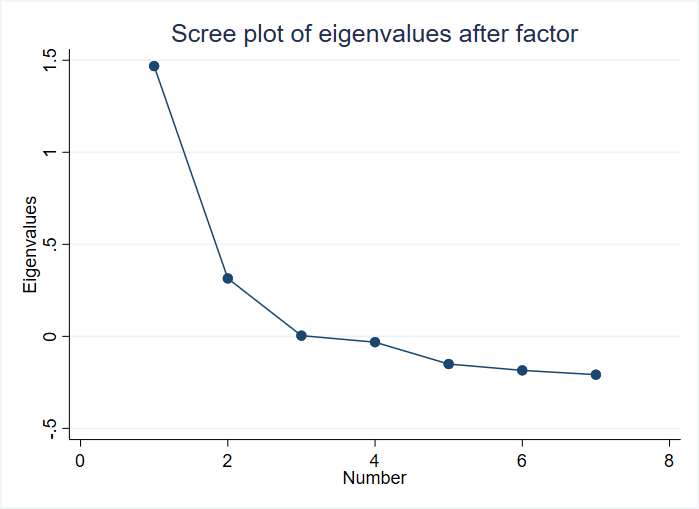
*

*Mokken analysis*

The Mokken analysis for the 7-item scale in presented in Table S1. The output of this analysis shows that the Loevinger H coefficients range between 0.11 and 0.38 with two items (6 and 7) below 0.30. This suggests that the items in the 7-item scale together possess ‘none’ to ‘weak’ scalability.

**Table S1 Mokken analysis presenting Loevinger H coefficients for the 7-item scale**

| Item | *H* |
| --- | --- |
| 1. Daytime frequency | .38 |
| 2. Nocturnal frequency | .32 |
| 3. Urgency | .32 |
| 4. Bleeding | .34 |
| 5. Well-being | .34 |
| 6. Arthritis | .11 |
| 7. Uveitis | .17 |
|  |  |

*Correlation analysis*

A correlation analysis for the 7-item scale presented in Table S2 suggests that no items were too highly correlated ( r > .50). However, there were non-significant correlations between items 6 and 7 and the rest of the scale (Table S2) suggesting that these items were measuring a trait independent of (not associated with) the rest of the items.

**Table S2. Pair-wise correlation analysis for the 7-item scale**

| Items | 1 | 2 | 3 | 4 | 5 | 6 | 7 |
| --- | --- | --- | --- | --- | --- | --- | --- |
| 1 Day freq. | - |  |  |  |  |  |  |
| 2 Night freq. | .44** | - |  |  |  |  |  |
| 3 urgency | .30** | .21** | - |  |  |  |  |
| 4 Bleeding | .36** | .29** | .27** | - |  |  |  |
| 5 Well-being | .26** | .25** | .33** | .23** | - |  |  |
| 6 Arthritis | .04 | .06 | .14** | .00 | .22** | - |  |
| 7 Uveitis | .02 | .07 | .07 | .07 | .19** | .12** | - |

*Note.* ***p* < .01

*Covariance residual coefficients correlation analysis*

A covariance residual coefficient correlation produced after the estimation of a CFA estimated across θ suggested that no pairs of items possessed residual coefficients over 0.20 (Yen, 1993). This suggests that no pairs of items violate local independence (no other *strong* dimensional trait), all residuals were between -.01 and .02.

**S4. Mathematical assumptions 4-item model**

*Factor analysis*

A factor analysis was conducted and the outcome suggested that there was a single major factor responsible for the majority of variance across the scale. A screeplot output is presented in Figure S1.

**Figure S2 Screeplot of the 4-item scale following a PCA analysis**


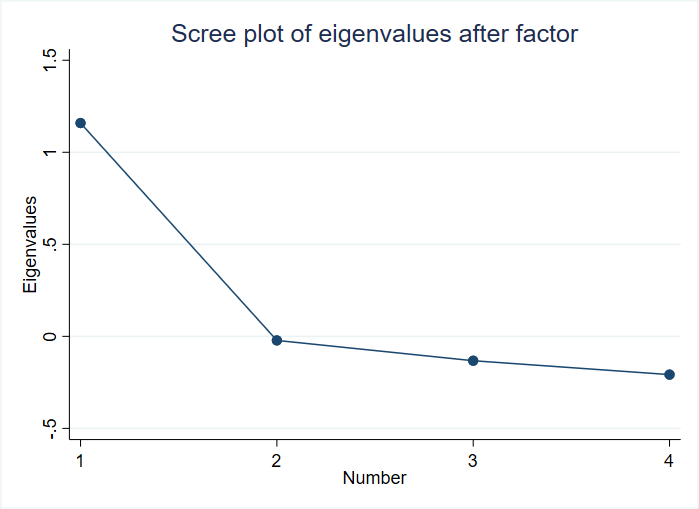


*Mokken analysis*

The Mokken analysis for the 4-item scale in presented in Table S4. The output of this analysis shows that the Loevinger H coefficients range between 0.41 and 0.48. This suggests that the items in the 4-item scale possess ‘medium’, bordering on high (.50 +) scalability.

| Item | *H* |
| --- | --- |
| 1. Daytime frequency | .48 |
| 2. Nocturnal frequency | .44 |
| 3. Urgency | .41 |
| 4. Bleeding | .43 |
|  |  |

**Table S3. Mokken analysis presenting Loevinger H coefficients for the 4-item scale**

*Correlation analysis*

A correlation analysis for the 4-item scale presented in Table S5 suggests that no items were too highly correlated (r > .50). All items were correlated with each other at *p* < .01 suggesting that these items were measuring a common trait (unidimensional)

**Table S4. Pair-wise correlation analysis for the 4-item scale**

| Items | 1 | 2 | 3 | 4 |
| --- | --- | --- | --- | --- |
| 1 Day freq. | - |  |  |  |
| 2 Night freq. | .44** | - |  |  |
| 3 urgency | .30** | .21** | - |  |
| 4 Bleeding | .36** | .29** | .27** | - |

*Note*. *P* < .01

*Covariance residual coefficients correlation analysis*

A covariance residual coefficient correlation produced after the estimation of a CFA estimated across θ suggested that no pairs of items possessed residual coefficients over 0.20 (Yen, 1993). This suggests that no pairs of items violate local independence (no other *strong* dimensional trait), all residuals were between -.01 and .01.
